# The role of farming and fishing in the rise of social complexity in the Central Andes: a stable isotope perspective

**DOI:** 10.1038/s41598-024-55436-4

**Published:** 2024-02-25

**Authors:** Luis Pezo-Lanfranco, André Carlo Colonese

**Affiliations:** 1 Institute of Environmental Science and Technology (ICTA), Universitat Autònoma de Barcelona, Cerdanyola del Vallès, Barcelona, Spain; 2 Department of Prehistory, Universitat Autònoma de Barcelona, Cerdanyola del Vallès, Barcelona, Spain

**Keywords:** Andean archaeology, Formative period, Archaic period, Stable isotopes, Bayesian stable isotope mixing models, Dietary reconstruction, Maize, Biogeochemistry, Ecology

## Abstract

For many years, the rise of stratified societies along the Central Andean coast, known as the birthplace of Andean civilization, has been closely linked to a marine-oriented economy. This hypothesis has recently been challenged by increasing evidence of plant management and cultivation among Andean populations long before the emergence of complex societies and monumental architecture. The extent to which marine and plant-based economies were integrated and their contributions to early sedentism, population growth, and intra-community stratification, however, remain subjects of ongoing and contentious debate. Using Bayesian Mixing Models we reanalyze the previously published stable isotopes (δ^15^N_collagen_, δ^13^C_collagen_, δ^13^C_apatite_) values of 572 human individuals from 39 archaeological sites in the Central Andes dated between ca. 7000 BCE and 200 CE to reconstruct dietary regimes in probabilistic terms. Our results reveal that fish, terrestrial fauna, and cultivated plants variably contributed to the diet of prehistoric Andean populations; in coastal and middle valley settlements plant cultivation, not fishing, fueled the development of the earliest complex societies during the Formative Period (from 3000 BCE). Similarly, in the highlands the societies that built ceremonial centers show a plant-based economy. Our findings also show that maize only became a staple food (> 25% dietary contribution) in more recent phases of Andean prehistory, around 500 BCE.

## Introduction

Over the past three decades, archaeological investigations have revolutionized our comprehension of the timelines and processes involved in early plant domestication along the Central Andean coast, a region renowned as the cradle of Andean civilization^1–4^. This transformation owes much to the meticulous analysis of plant micro- and macro-remains, which have provided invaluable insights into the chronological sequence and possible routes through which domesticated crops were disseminated throughout the region^5–8^. Studies indicate that during the Late Pleistocene, Early Holocene, and Middle Holocene periods, prehistoric populations in the Central Andes subsisted by gathering a diverse array of terrestrial and marine fauna. Concurrently, they initiated the management and cultivation of semi-domesticated and domesticated plants as early as 8350 BCE^9,10^. These food production systems were complemented by the development of irrigation technologies as early as 4550 BCE^11^, and camelid domestication (between 6500 and 3500 BCE)^12^, leading to deep economic and social changes.

Remarkably, a comprehensive review and calibration of available radiocarbon dates associated with food remains spanning from the Late Pleistocene to the Inca period have unveiled that among the 51 plants used during the time of European contact, at least 40 were under cultivation during the preceramic era, predating approximately 1800 BCE^12^. Overall, the emerging evidence suggests that the temporal gap between the early adoption of cultivated plants and the emergence of “full-fledged agriculture” (sensu Zeder^13^, an economic system primarily based on the production and reliance upon domesticates) may not have been as protracted as previously assumed^,^^14,15^, indicating that preceramic societies responsible for the early monumental centers likely practiced agriculture.

Other lines of evidence also support this hypothesis. Studies of demographic dynamics in precontact South America detected a period of exponential population growth starting around 3000–2000 BCE, linked to environmental changes and the expansion of farming systems. In the Central Andes, an increase in settlement density suggests that population growth rates were particularly high between 2000–1 BCE^16^. This is consistent with other empirical evidence for population increases during the Formative period (3000–1 BCE), including larger size and agglutinated distribution of settlements, as well as changes in domestic architecture^17–19^.

For the Peruvian Coast, however, the role of fishing and farming in the processes of social complexity, reflected in monumental architecture and ceremonial centers, sites hierarchization, and intracommunity stratification, which in turn may reflect the existence of large scale (regional) social cooperation or control of labor, and possibly the existence of centralized institutions^1,2,15,19,20^ has been widely debated^15,19–22^. Seminal works emphasized the marine economy as the key factor in the development of sedentism, population growth, and social stratification in the region, suggesting that social divisions would have arisen around an early centralization and strategic distribution of marine production, which would have “pre-adapted” coastal groups to a later increasingly hierarchical development based on relatively late agriculture. In this scenario, agriculture was introduced long after the initial complexity, approximately 1800 BCE, as a way of maintaining the earlier institutionalized social structures^15^.

Later discoveries of older and even larger monumental sites in the middle valleys have, however, reinforced the hypothesis that civilization arose inland based on an agricultural economy, rather than on the coastline. At least 35 planned sites integrated into intra-valley hierarchical systems in the North-Central Coast of Peru, with over 200 radiocarbon dates between 4100–1800 BCE, have led archaeologists to rethink the origins of urbanization processes and re-examine the chronology of the Central Andes as a whole^1,23^. Additionally, evidence of macro- and micro-botanical remains shows that coastal economies were more diverse and richer in domesticated plants than previously considered^24–26^. Even in models that minimize material factors as “triggers” of social stratification and emphasize behavioral causes, such as power accumulation strategies and manipulation of belief systems, the existence of subsistence systems able to support increasing populations in sedentary settlements would have played a critical role^27,28^.

According to recent discoveries, since at least the Initial Formative Period (3000–1 BCE) the communities of the Pacific basin valleys would have been organized around symbiotic farming-fishing economies, with trade networks along transversal littoral-inland corridors that would not typically exceed 80–100 km^29^. Coastal societies would have relied primarily on fishing with some dependence on cultivated products, while communities in the middle and higher sectors of the valleys cultivated semi-domesticated or domesticated plants supplemented by marine products such as dry-salted fish and mollusks^11,29,30^. In the highlands, the archaeological record of ceremonial centers suggests that subsistence was largely based on terrestrial food items^31–33^. However, the relative contribution of plants and terrestrial and marine protein sources to diets at different elevations and latitudes is not well understood, and their importance to the process of social complexity remains controversial^29,30,34,35^.

In addition, the paths of the agricultural intensification of certain plants and their role in sustaining dense populations and social structures during different stages of the process is still in discussion^26,30,36^. Among these, maize (*Zea mays*) is of particular interest. Maize began its domestication process around 9000 years ago in the Mexican lowlands^37^ and spread into South America around 6000 BCE where it evolved isolated from the wild teosinte gene pool^38^. It reached the Peruvian North Coast as early as 4800–4500 BCE^8^, possibly through multiple waves of colonization^39,40^. Until recently, the Central Andes was considered a “2nd center of domestication for maize”^36^, but currently is recognized as an “improvement center” where local adaptations of partially domesticated maize occurred^38^. While maize’s importance as a staple food has been demonstrated in later periods, linked to state and empire domination strategies (e.g., as maize beer offered during community work, feasts, and ceremonies)^12,41,42^, its significance as a prime mover of the earliest complex societies remains unclear^30,31,43,44^.

Available evidence suggests that its consumption may have been limited in the region, possibly only in ceremonial contexts or as a seasonal crop, until later times^45,46^. Stable carbon and nitrogen isotope analyses of collagen (δ^13^C_coll_ and δ^15^N) and bioapatite (δ^13^C_ap_) from exhumed human bones and teeth have proven to be an unparalleled method to study subsistence in past populations^47^. The δ^13^C_coll_ is a proxy for dietary protein sources and photosynthetic pathways of plants in the food web (C_3_, C_4_, CAM). The δ^13^C_ap_ instead reflects the isotopic composition of all macronutrients (carbohydrates, lipids, proteins) in an individual’s diet, offering insights into the energetic constituents^48^. The analysis of δ^13^C_coll_ and δ^13^C_ap_ can effectively discriminate diets based on C_3_ from C_4_ (i.e., maize and amaranth, a high altitude plant^12^) and CAM plants (cactuses with isotope values that overlap C_4_ in the Andean case) plants due to fundamental differences in their isotopic composition^49,50^; however, they are not suitable for distinguishing between diets based on marine resources and those based on C_4_ plants, due to overlapping values^51^. This limitation is usually addressed by analyzing the δ^15^N value since it is affected by relatively well-established trophic fractionations (3–6‰), which are larger in aquatic systems due to longer and more complex food webs compared to terrestrial systems^52^. High δ^15^N values, however, can be also related to aridity^53^ and fertilization practices (e.g., camelid dung, seabird guano)^54,55^, two factors to be considered in isotopic analyses in the Central Andes.

Stable isotope analyses have been successfully employed to reconstruct diets in prehistoric Andean populations, particularly for later periods, during which the main dietary trends and shifts have been identified^17^. However, the evidence for earlier periods, when the first complex societies arose, is more limited^30,34,44,46^. Despite the excellent morphological preservation of human remains and the growing interest in the relationship between subsistence systems and social changes, efforts to understand dietary shifts at the individual or population level have been hindered due to the lack of viable collagen from prehistoric populations beyond the first millennium CE^30,56–58^. Furthermore, until recently, the analytical methods used only allowed for a qualitative approach to the dietary constituents, which did not clarify in quantitative terms the contribution of C_3_ plants (e.g., tubers, legumes) and C_4_ plants such as maize, nor the contribution of marine and terrestrial proteins in these early diets.

**Figure 1:**
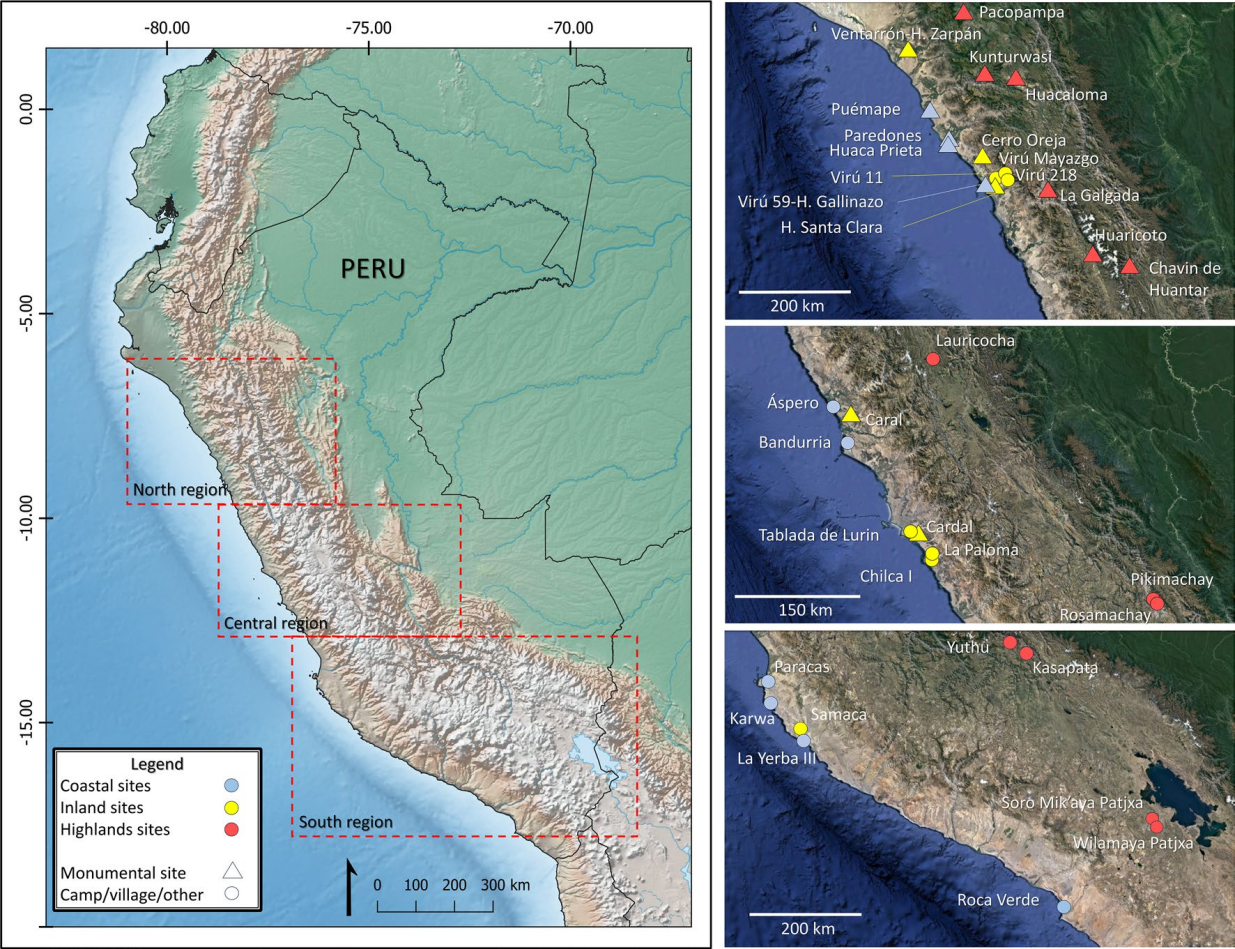
Location of the archaeological sites mentioned in the text and tables. Map generated with QGIS v. 3.2.8 (https://www.qgis.org) with images of Google Earth Pro® (2023 Google LLC. All rights reserved) and a raster basemap from https://www.naturalearthdata.com/.

Here, we reassess the stable carbon and nitrogen isotope values of 572 pre-Columbian human individuals from Peru. These individuals represent 61 populations dated between ca. 7000 BCE and 200 CE, through the Archaic (7000–3000 BCE), Formative (3000–1 BCE) and Early Intermediate (1–600 BCE) periods, and were recovered from 39 archaeological sites located along the coast, middle valleys, and highlands of the three distinct latitudinal regions (i.e., North, Central, and South) of the Central Andes, characterized by contrasting environmental conditions (Fig. 1, see Supporting Information 1). We also included isotope values from 408 faunal and 404 plant specimens discriminated by subregion, to characterize regional and local food webs (Supporting Information 2).

A Bayesian stable isotope mixing models (BSIMMs) approach was used to estimate the proportional caloric contributions of marine and terrestrial fauna, as well as C_3_ and C_4_ plants, to the diets of individuals from different sites and periods^30,59–61^ to assess their economic importance for Andean communities over the Archaic and Formative periods and refine the chronology of major dietary shifts. Out of 572 individuals, 293 individuals with confident parameters for collagen preservation^62,63^ and a complete set of isotope values (i.e., δ^13^C_coll_, δ^15^N, and δ^13^C_ap_) were included in the BSIMMs reconstructions (see Material and Methods and Supporting Information 3).

Our aim is to determine whether major social changes, demographic growth, and political-ideological transitions, which usually define the archaeological periodization, are linked to the intensification of key crops, intended as the substantial increased intake of domestic plants. While our primary focus is to elucidate the role of marine and terrestrial resources on the Andean coast, highland diets have been included to document different trajectories in crop adoption along this vast and heterogeneous environment.

## Results

### Stable isotope analysis and Bayesian dietary reconstruction

**Figure 2:**
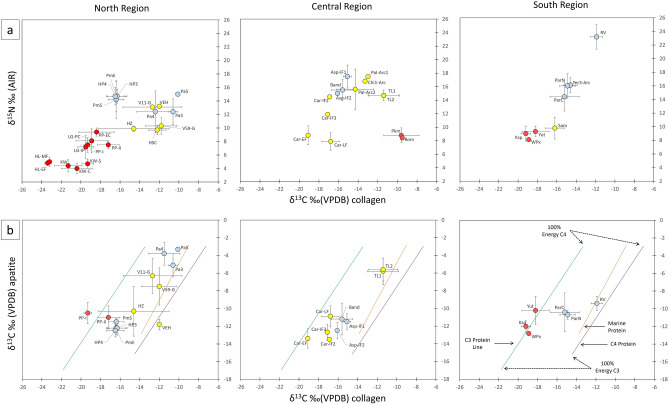
(**a**) Average δ^13^C_coll_ and δ^15^N values from prehistoric populations of the Central Andes according to region. (**b**) Average δ^13^C_coll_ and δ^13^C_ap_ values from human individuals in the workspace of Kellner and Schoeninger^48^, discriminated by region for comparative purposes. In this model, the regression lines represent the source of protein (C_3_, marine, and C_4_) and the location of the values along the lines indicate the relative proportion of energy provided by the source. Red dots represent highland populations. Dots represent averages and crosses are standard deviations (1σ).

In the available dataset, human individuals had δ^13^C_coll_ values (n = 317) ranging from − 23.8‰ to − 9.1‰, δ^15^N values (n = 294) from + 2.8 ‰ to + 24.9 ‰, and δ^13^C_ap_ values (n = 400) from − 15.7 ‰ to − 2.4‰. The δ^13^C_coll_ and δ^15^N values of coastal populations fall within the range of expected values for marine based diets, notably during the Archaic period (7000–3000 BCE), whereas inland and highland populations show values consistent with diets based on C_3_ plant ecosystems (Fig. 2a). Some populations, however, show wide standard deviations suggesting quite diverse diets. As expected, human δ^15^N values decrease significantly with increasing distance from the sea (n = 292; Spearman’s ρ =  − 0.693; *p* < 0.01).

The δ^13^C_coll_ and δ^13^C_ap_ values (Fig. 2b), which track the sources of protein and energy components of the diet, respectively, show mixed subsistence strategies and three different dietary regimes through time: 1) the diets of the Initial, Early and Middle Formative populations (3000–600 BCE) with a significant contribution of C_3_ plants (i.e., legumes, tubers, and cucurbits) and a more discrete contribution of C_4_ plants (maize and potentially amaranth, depending on ecological setting); 2) the diets of Late Formative populations (600–1 BCE) based on C_3_ plants with a growing consumption of C_4_; and 3) diets based on a higher intake of C_4_ plants and less C_3_ plants, mostly linked to populations dated between 200 BCE and the first centuries of the current era.

It is worth noting that while C_4_ plant intake varied considerably among early populations (for instance, an early dependence on C_4_ plants appears evident in the North Coast since at least 4500 BCE^44^), their consumption increased significantly in later populations of the North and Central coasts, especially during the last half of the first millennium BCE (*p* < 0.05 with Kruskal–Wallis-H for δ^13^C_ap_; see post-hoc tests in Supporting Information 1: SI1 T4).

**Figure 3:**
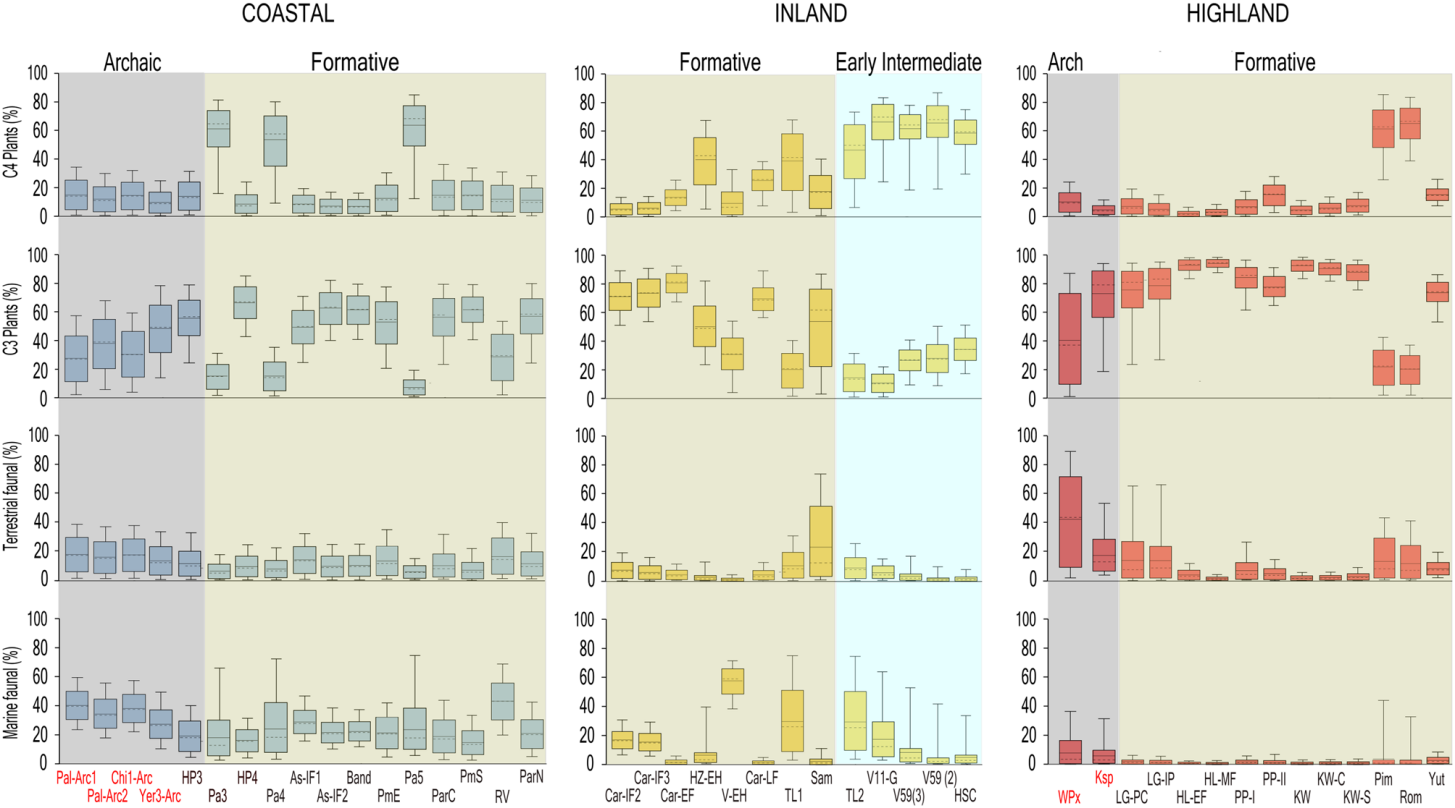
Dietary caloric estimations of food sources in Andean populations grouped by geographic regions, and archaeological periods: Archaic (Arch); Formative; Early Intermediate. The boxes represent a 68% confidence interval (between the 16th and 84th percentiles) and the whiskers a 95% confidence interval (corresponding to the 2.5th and 97.5th percentiles). The horizontal discontinuous line indicates the median (50th percentile). The horizontal continuous line indicates the mean. Sites’ IDs in black refer to sites or phases with monumental architecture or hierarchical distribution of settlements. IDs in red refer to non-complex sites (see also SI1 and SI4 F1and F2).

In conclusion, estimates from BSIMMs indicate that, in general, C_3_ plants were the main sources of dietary calories for most of the populations during the Formative periods, and notably in the highlands. Marine resources provided most proteins to coastal populations, decreasing inland and in the highlands. Calories from maize, instead, increased over time, notably among coastal and inland populations from the North and Central regions during the Late Formative and the Early Intermediate (Fig. 3; see also Supporting Information 4). However, we acknowledge that his interpretation is mostly based on five outlier populations, thus, further studies are required to confirm this hypothesis.

## Discussion

### Regional dietary trends

In the Central Andes, the role of agriculture and fisheries as economic drivers for sedentism, population growth and social stratification has been widely debated, and a wide variability in diets across time periods is observed in different subregions. Coastal and highland populations show more reliance on marine products and C_3_ plants, respectively. However, coastal diets show also high reliance on C_3_ plants. Among inland groups, a notable overlap of isotope values suggests more diverse diets based on plants.

On the North Coast, maize was an important dietary item at the site of Paredones as early as 4500 to 4000 BCE, whereas in the neighboring site of Huaca Prieta people relied on marine foods complemented with terrestrial items and a little maize around 3000–2500 BCE^8,10,44,64^. From the Early Formative (1200–1000 BCE) to the Salinar period (400–200 BCE), communities flourished with a combination of C_3_ plants, marine and terrestrial protein, with low C_4_ contribution (< 15%). Archaeological inventories and coprolite analyses indicate diets of C_3_ plants (i.e., tubers, beans, peanuts, and carob), fish (e.g., *Squatina armata*, *Mustelus* sp., *Paralonchurus* sp., *Cynoscion* sp., *Sciaena deliciosa*), marine mammals (*Otaria* sp.) and shellfish (e.g., *Mesodesma donacium, Choromytilus chorus, Polinices uber, Thais* sp.), and some maize^57,65^. Increasing maize contribution, making up more than 60% of the dietary calories, is clear in inland sites (Viru 11, Viru 59) around 200 BCE–200 CE, during the Gallinazo period, between the Late Formative and the Early Intermediate^57,66^.

In the Northern highlands, diets were dominated by C_3_ plants over the evaluated periods (Fig. 3). This scenario of C_3_ preference (i.e., quinoa, beans, and various tubers) can be extended regionally to other Formative sites with monumental architecture, such as Huaricoto (~ 2200–200 BCE), Huacaloma (1500–550 BCE), Pacopampa (900–600 BCE), Chavin (850–450 BCE), and Kuntur Wasi (800–1 BCE). Maize increases slightly only at the beginning of the current era^31–33,46,56^. Terrestrial protein was potentially more important at early sites, like La Galgada, possibly due to easily accessible cervids or camelids^32^. Later, diets were low protein, despite being herders, like in Pacopampa^33^.

On the Central Coast, Archaic populations from La Paloma and Chilca I show the greatest dependence on marine and terrestrial fauna among all the compared groups^34,67^ (Fig. 3). Although macro- and micro-botanical remains (phytoliths, starches, and pollen) from Fortaleza, Pativilca, and Supe valleys dated between 2900 and 2100 BCE, confirm maize consumption during the Initial Formative^6,25,26^, isotopic analyses suggest that its consumption was limited and sporadic, with Formative sites instead showing a reliance on C_3_ plants. For instance, Caral individuals during the Initial Formative showed dietary calorie contributions of 62% C_3_ plants and less than 7% maize, while diets from Áspero and Bandurria^30,68^, two early settlements with monumental architecture located on the shoreline, had contributions of 50–60% C_3_ plants. C_3_ plant diets predominated the Early and Middle Formative, but these preferences changed in the Late Formative with maize contributions increasing by 10% to 25%^30^. For example, in Tablada de Lurin (200 BCE–100 CE), based on combination of C_4_ plants and marine resources, maize provided around 40–50% of dietary calories^56,58^.

In the Southern region, during the Late Formative period, individuals from Paracas communities (550–100 BCE), had a diet composed of C_3_ plants (56–57% of dietary calories), mainly tubers (manioc, *jíquima, achira*, among others^69^), marine resources (19–21% of dietary calories), and maize (11–14% of dietary calories). Individuals from Samaca cemeteries (100 BCE–100 CE) in the lower Ica Valley, show dietary preference for C_3_ plants (i.e., peanuts, squash, manioc, and *pacay*) and low amounts of C_4_ plants (i.e., maize, and possibly CAM plants), and restricted marine foods despite a relatively short distance to the sea^70^. In this region, maize intensification seems to have been delayed despite the proximity to Ayacucho and Cusco in the South Highlands, where maize cultivation was hegemonic at least since 800 BCE^71,72^.

The compiled isotope data reveals dietary trends during the Archaic period (7000–3000 BCE), showing that coastal individuals had diverse diets primarily composed of marine resources and C_3_ plants (e.g., tubers, cucurbits, and legumes), alongside terrestrial mammals, which aligns with faunal remains^30,67^. In contrast, highland individuals primarily relied on C_3_ plants and terrestrial mammals during this period. Moving into the Formative Period (3000–1 BCE), coastal, inland, and highland populations increasingly incorporated C_3_ plants into their diets, although coastal communities continued to consume fish. Maize played a limited role during the Formative Period, with notable exceptions like Paredones. Maize consumption saw a notable rise during the first millennium BCE, a period linked to increasing population pressure^16,17,30^, contributing to approximately 25% of calories in some inland sites by 500 BCE. Its intensification was more marked on the North Coast (between 400–100 BCE) and the Central Coast (~ 200–1 BCE), and slower on the South Coast (between 750–450 BCE)^73^. It eventually became a staple between 200 BCE and 200 CE in the North and Central coasts. This increase in maize production and consumption during the last half of the first millennium BCE is probably linked to *chicha* production for ceremonial activities^74^ and is the precursor to the extensive production and use of *chicha* in Early Intermediate Period and Middle Horizon contexts^75^.

While archaeological and botanical evidence strongly supports maize as the primary C_4_ plant in the Andes, it is important to note that maize was not the exclusive source of ^13^C-enriched values in Andean diets. The overlapping δ^13^C values of maize, amaranth, and CAM plants (i.e., cactuses) is a confusing factor that should be considered in the interpretation of maize adoption in arid and high-altitude environments. However, the available evidence suggests that cactuses were more important for Archaic populations, and amaranth, as staple, possibly was restricted to highlands or lower latitudes^12,70,76^.

This extended process of farming intensification among Andean communities involved the implementation of productive strategies that were well-suited to the specific crops, soils, and limited water resources in these arid and challenging landscapes (i.e., subterranean water management, increasing irrigation technology, sunken fields, flooding agriculture, consortium cultivation etc.^77^), modulated by changing environmental conditions^16,78–80^. These technological improvements probably did not occur at the same time and not necessarily were related to the same factors in each subregion. Social and political structures could have variably affected the way that crops were adopted and used by populations from different altitudes, characterizing particular processes for each region and site.

The factors that conditioned maize intensification by a few “maize-dependent” populations and preclude its adoption by most Formative communities are possibly related to ecological (e.g., water availability, land features, climate, and altitude restrictions) and cultural (e.g., identity) circumstances. For instance, the wider valleys of the North Coast, especially those with permanent water supply are more appropriate for maize cultivation with insipient technology^20^.

To conclude, this analysis helps address three foundational questions about the diet in the Archaic and Formative periods of the Central Andes: 1) the diet that led to the development of societies linked to monumental architecture, presumably complex societies, was not predominantly marine in caloric terms; 2) these developments were based mainly in C_3_ plants at any altitude, including coastal sites; and 3) most diets were not based on maize, which, in turn, was a crop of marginal importance at least until the first millennium BCE. Thus, in accordance with previous arguments, for coastal settings, our results challenge the prevailing theory that marine resources were the primary economic driver for social complexity. Our results, instead, align well with the emerging view that political development in most inland and highland sites relied on productive systems based on C_3_ crops. It emphasizes the significance of plant cultivation in propelling population growth, social stratification, and the development of political institutions in one of the cradles of civilization.

## Methods

### Archaeological sites and individuals

This is an *in-silico* study developed using previously published stable isotope data hosted in the South American Archeological Isotopic Database (SAAID_V.2.0_2023, hosted in CORA Platform—10.34810/data602). A total of 572 human individuals from 39 archaeological sites of the Central Andes, which represent the totality of the available data before the current era, were included in our analysis.

The database with raw isotope values of individuals (consumers’ values) is reported in the Supporting Information 1. Sex and age estimates are reported as in the original references. Most of the sites are in the Peruvian valleys that transversally cross the Pacific Coastal Desert to drain in the Pacific Ocean, at coastal (i.e., less than 3 km from the sea; n = 10), and inland (i.e., lower, and middle valleys < 600 m above the sea level, located at any distance from the coast; n = 16), locations. Other sites (n = 13) are in the Andean highlands (i.e., upper valleys of the Pacific Basin with altitude > 600 masl, and interandean valleys at any altitude). Because some of the sites have long occupational sequences, a total of 61 discrete populations were analyzed (see Supporting Information 1). Sites and phases were classified by social complexity in two categories: 1) sites with monumental architecture or ceremonial centers, which correspond to more or less complex societies; and 2) other type of sites (camps, towns, open sites, rock shelters), that possibly correspond to non-complex or egalitarian societies. This classification was contrasted with the archaeological interpretations for each site based in several markers (i.e., architectural features, construction volume, intravalley settlement patterns, mortuary evidence, and archaeological inventories) and corrected when necessary (e.g., Paracas Peninsula sites, which are tombs linked to a complex settlement network and rich mortuary offerings).

The chronological framework of the sites and cultural phases were based on re-calibrated radiocarbon dates provided in the references (see details in Supporting Information 3). The time span of examined individuals comprises the Archaic (7000–3000 BCE), Formative (3000–1 BCE), and Early Intermediate (1–600 BCE) periods.

Our analysis also included isotope values from 812 specimens of fauna (n = 408) and plants (n = 404) for food web reconstructions (see Supporting Information 2). These values were discriminated by latitudinal region to generate more reliable inputs for Bayesian models and visual interpretations. An isotopic baseline of potential dietary sources by each sub-region was generated with rKin using Standard Ellipse Areas^81^ (see Supporting Information 3). All the isotope values were extracted from SAAID.

To prevent the potentially confusing effect of taphonomy issues in our analysis, only individuals with reliable δ^13^C_coll_ and δ^15^N values from bone and dentine collagen, according to accepted preservation criteria^62,63^, were included. For δ^13^C_ap_ values, although the volume of published data is higher than collagen, preservation criteria have not been used extensively, especially in older studies, and the assumption of reliability has been largely based on the extraction protocols^82^. In this research, we used bone *δ*^13^C_ap_ values only if a sample’s collagen was well-preserved. We assumed that if collagen, an organic biomolecule that forms the biological structure housing inorganic crystals, is well-preserved, then bioapatite is also well-preserved. However, apart from data consistency (the values in apatite are typically ^13^C-enriched compared to collagen), there are no other objective markers to assess apatite preservation^83^. Because dental enamel preserves better than bone, all available enamel δ^13^C_ap_ values from selected individuals were employed.

To avoid confounding factors of breastfeeding and weaning, just adults with acceptable collagen atomic composition were included in our analyses. Infants and toddlers (subadults < 4 years) were not included^84^. According to these criteria, 293 human individuals with confident parameters for collagen preservation and a complete set of isotope values (i.e., δ^13^C_coll_, δ^15^N, and δ^13^C_ap_) were selected for BSIMMs analyses.

### Isotopic analysis and BSIMMs

First, we used descriptive statistics and conventional isotope scatterplots as diagnostic tools to identify major dietary trends and shifts for each population and region. BSIMMs were then run for each group. Scatterplots of δ^15^N vs. δ^13^C_coll_ provide information about the type of protein consumed, terrestrial (nitrogen below + 11 ‰ and carbon below − 12.5 ‰), or marine if both values are very high^52^. Scatterplots of *δ*^13^C_coll_ vs. *δ*^13^C_ap_ in a workspace of three regression lines derived from the experimental feeding studies, allows for discrimination of the protein and carbohydrate (C_3_ or C_4_) sources. The distribution of an individual’s values on the axis of the apatite indicates if the energy of the diet is C_3_ or C_4_, and in the axis of collagen, if the protein source is C_3_ or C_4_/marine. Populations concentrated between the two lines would have complex diets, combining C_3_ and C_4_ proteins. The distance to the end lines indicates the approximate proportion of C_3_ or C_4_ energy consumed^48^.

Descriptive statistics (mean, range, standard deviation, standard error of the mean) and comparisons of isotope values between groups, were used to detect diagnostic differences over time and space. These analyses were performed using Kruskal–Wallis tests (α = 0.05), after evaluation for normal distribution with Kolmogorov–Smirnov test for normality (α = 0.05). Mann–Whitney U (α = 0.05) was used as a post-hoc test, accordingly. Spearman’s correlation was used to test the relationships between isotope values and distance to the sea. All the tests were performed in SPSS v.21 (Microsoft®).

The wide range of dietary variations in macronutrient composition, isotopic values of consumed items, fractionation (diet-to-consumer offsets), and nutrient routing factors, along with other sources of uncertainty, often hinder accurate dietary reconstructions using graphic or linear methods alone. In contrast, BSIMMs allow for the inclusion of multiple proxies and sources of variation, enabling more reliable estimates of dietary composition as probability distributions^59–61^.

The software *Food Reconstruction Using Isotopic Transferred Signals* (FRUITS v.2.1.1 Beta program), was used to estimate the proportional calorie contribution of different food sources to human diets, based on stable isotope values as dietary proxies^59^. A weighted model with fractionation factors (offsets) based on nutrient concentrations in the respective food groups was applied^60,61^.

The *consumer data* for individuals consist of their isotopic values with an uncertainty of ± 0.5^60^. For groups, we used the average isotope values of each period/site and the standard error of the mean*.* After a careful screening of preservation criteria for collagen in the SAAID database, only individuals with valid collagen isotope values from each period/site were included in the analysis. When available, a complete set of δ^13^C_coll_, δ^15^N_coll_ and δ^13^C_ap_ values was included in models of three proxies. However, because some individuals/populations had only δ^13^C_coll_, δ^15^N_coll_ values, we were required to implement alternative models with two proxies for comparative purposes. Two-proxy models can reflect the main trends in dietary compositions but may be less confident in the exact proportions of the analyzed food sources compared to three-proxy models^30,59,60^.

The potential assimilated food sources (*Food groups*), with their respective composition of macro-nutrients (*Food fractions:* bulk, protein, and energy) were grouped in four categories: terrestrial faunal (providing proteins and lipids), called “TF” in the model; marine faunal (providing proteins and lipids), called “MF”; and C_3_ and C_4_ plants (providing carbohydrates and proteins), called C_3_ and C_4_, respectively^30,59–61^.

The isotope values from each food group come from isotopic paleodietary studies focused on the Central Andes valleys classified in three latitudinal regions: the Central Coast and Central Highlands, the North Coast and Northern Highlands, and the South Coast and Southern Highlands. For consistency, each site was analyzed in accordance with isotopic data from regional or local species, paying special attention to a site’s archaeological inventories or previous isotope values from local modern or archaeological samples. Although this implies the omission of some valuable references, each region was characterized with its own values. If local or regional values were not available, values from neighboring regions were used. For comparisons with the archaeological values, δ^13^C values from modern specimens were corrected for the fossil fuel effect, adjusting the values by + 1.5 ‰^85^.

For marine fauna, a synthesis of several sources was used, mainly based on modern data from the Central Coast^30^. Only isotope values from modern specimens were used for plants to avoid issues related to preservation^86,87^. The average δ^13^C and *δ*^15^N of food groups, the number of specimens, and species included in the models, as well as the original reference of the values for the Central, North, and South regions, can all be found in the Supporting Information 2.

Isotope fractionation factors are consensual values derived from experimental studies. For δ^13^C, the fractionation factor was set at + 4.8 ± 0.5 ‰ between diet and collagen, and + 10.1 ± 0.5 ‰ between diet and apatite. The δ^15^N diet-collagen fractionation factor was set at + 5.5 ± 0.5 ‰^59–61,88^.

The weighted values of each fraction of macronutrients (lipids, carbohydrates, and proteins) were set based on previously published parameters^30,59–61^. The values of δ^13^C_ap_ represent the total carbon mix in the diet. Therefore, we use the same bulk value for each food group^56–58^. Terrestrial and marine fauna δ^13^C bulk values were estimated as a weighted mean of lipid and protein δ^13^C values^59,60^ For collagen, the carbon of the protein and energy routed to the total collagen was established at 74 ± 4% and 26%, respectively^89^, although carbon can be routed from carbohydrates and lipids recycled during the synthesis of non-essential amino acids^90^. We assumed that nitrogen was derived exclusively from proteins (100%). Lipids and carbohydrates were added to the model as “energy”. In order to integrate this combinatory effect, we applied a “concentration-dependent” model^59–61^.

Following previous reconstructions using FRUITS^30,59–61^, the isotopic composition of each nutritional fraction (protein, carbohydrates, and lipids) was obtained from the mean values of δ^13^C_coll_ and δ^15^N_coll_ using the following fractionation factors: − 2‰ (*∆*^13^C_protein-collagen_), − 8‰ (*∆*^13^C_lípids-collagen_), and + 2‰ (∆^15^N_protein-collagen_) for terrestrial mammals, and − 1‰ (*∆*^13^C_protein-collagen_), − 7‰ (∆^13^C_lípids-collagen_), and + 2‰ (∆^15^N_protein-collagen_) for marine animals. For plants, the offsets were − 2‰ (∆^13^C_bulk-protein_) and + 0.5‰ (∆^13^C_bulk-lipids_), while for the δ^15^N value of plant protein, the known value of δ^15^N recorded for the plant was assumed. The carbon weight (*concentrations*) of each food fraction (protein and energy) from each food group was calculated according to its macronutrient composition^30,59–61^ (see details in Supporting Information 3).

Finally, the dietary estimates were parametrized by a physiological, conservative, and acceptable range of protein consumption stipulated between 5 and 45% of the total calories^59^. This input was charged as “prior”. Guided by our observations of the bivariate plots, alternative models were tested for each site and population. The estimates generated for FRUITS models reflect carbon content or equivalent calorie contributions and are expressed as relative contributions (adding up to 1 or 100%) with an associated 1σ uncertainty^60^. The estimations of food group contributions are reported in the Supporting Information 4 (see also Supporting Information 5).

### Study limitations

It is necessary to objectively judge the limitations of this study and acknowledge the inherent complexity of economic and sociocultural processes in the Andean region. These processes go beyond what can be captured through dietary reconstructions alone. As new research emerges, the current understanding of the topic may evolve, and we must remain open to revising our interpretations based on additional findings.

In addition, this study uncovered some complicating factors that may have affected the overall data interpretations: 1) information to asses bone collagen preservation according to established collagen quality criteria^62,63^ were not always available in the surveyed literature; 2) several ancient Andean populations have poor collagen preservation, thus not providing sufficient bone collagen for dating or isotopic analyses, and considerable temporal and regional gaps were observed in the sampled isotope data; 3) the sites contained different numbers of human individuals, which may impact the observed variability; 4) some sites lack information on faunal and botanical remains, and the data preclude the construction of local isotope baselines therefore limiting the possibility of obtaining refined dietary estimates; 5) most of studies from the past three decades included in this research did not have access to the methods currently used for analyzing bioapatite integrity (i.e., Raman spectroscopy and FTIR)^83^, thus, enamel and bioapatite diagenesis cannot be estimated, additionally, there is a potential issue with the interlaboratory variation of δ^13^C_ap_ analysis^91^; 7) despite the accuracy of the methods employed, we should consider isotopic equifinality, that is, varying combinations of food contributions that may result in the same consumer isotopic values; finally, 8) isotopic markers are unable to differentiate cultivated from non-cultivated plants, which is central in this discussion, however, based on available references^2,4,7,10,12,26^, we can assume, that most plants behind the isotopic values observed in this study were domesticated crops.

### Supplementary Information

Supplementary Information 1.

Supplementary Information 2.

Supplementary Information 3.

Supplementary Information 4.

Supplementary Information 5.

## Data Availability

All data that support paper’s conclusions are present in the main text and the Supporting Information files. The stable isotope data used in this research have been previously published and were obtained in compliance with the regulations of the Peruvian government (i.e., *Ministerio de Cultura* since 2010 and, previously, by the *Instituto Nacional de Cultura*). This research did not involve direct processing of human tissues, and there are no ethical issues related to the handling of published data.
