# Supplementary material for: The role of farming and fishing in the rise of social complexity in the Central Andes: a stable isotope perspective
Source: Sci Rep. 2024 Feb 25;14:4582. doi: 10.1038/s41598-024-55436-4 (PMC10894859; doi:10.1038/s41598-024-55436-4)
Supplement: Supplementary file 3 — Supplementary Information 3. [file 41598_2024_55436_MOESM3_ESM.docx]

**Supporting Information 3**

**Radiocarbon calibration**

The chronological framework of archaeological sites and cultural phases was based on the periodization proposed by the original reference. When available, radiocarbon dates, direct (mainly AMS dates) or derived from associated materials reported in the references were re-calibrated (BCE/CE) using a combination of the marine (Marine20, Heaton et al., 2020) and terrestrial (SHCal20, Hogg et al., 2020) curves in OxCal v.4.3 (Bronk Ramsey, 2009) according to material dated. We estimated the local marine radiocarbon reservoir correction value (∆R) of each site using data from the Marine Reservoir Correction Database (Reimer and Reimer 2001, see <http://calib.org/marine/>). For individuals directly dated, we included the mean relative contribution of marine carbon to collagen estimated with FRUITS. Calibrated dates were rounded to 10. Then, populations were classified by archaeological period.

**Mean values of food groups from Central Andes**

Our analysis included isotope values from 812 specimens of fauna (n = 408) and plants (n = 404) for food-webs reconstructions. The isotope values from each food group come from isotopic paleodietary studies focused on the Central Andes valleys classified in three latitudinal regions, the Central Coast and Central Highlands, the North Coast and Northern Highlands, and the South Coast and South Highlands. For consistency, each site was analyzed in accordance with isotopic data from regional or local species, paying special attention to site’s archaeological inventories or previous isotope values from modern or archaeological local samples. Each region was characterized with their own values. If local or regional values were not available, values from the neighbor regions were used. For comparisons with the archaeological values, *δ*^13^C values from modern specimens were corrected for the “fossil fuel effect” adjusting the values by +1.5 ‰ (Marino and McElroy 1991).

An isotopic baseline of potential dietary sources by each sub-region was generated with rKin using Standard Ellipse Areas (Albeke 2017; Robinson et al., 2022) for comparative purposes (Figure SI3 1).

In the Central Andes as a whole, C_3_ plant (including tubers, legumes, annuals, and fruits) predominate and have *δ*^13^C and *δ*^15^N mean values of −24.6 ± 1.6‰ and +3.8 ± 3.5‰, respectively. Among the C_4_ plants, there are only two domesticated crops (e.g., the highlands’ crop *Amarantus* sp., and *Zea mays*, which grows in several temperate environments), with *δ*^13^C and *δ*^15^N mean values of −10.9 ± 1.2‰ and +6.4 ± 3.4‰, that clearly overlap with CAM (Crassulacean Acid Metabolism) plants such as cacti, which have *δ*^13^C and *δ*^15^N mean values of −10.9 ± 0.4‰ and +4.4 ± 0.7‰ (*86-88*). Although some variability of isotope values in plants from Central Andes can be linked to arid conditions and/or the use of fertilizers (e.g., camelid dung, seabird guano) (*89-90*), an overview of our entire set of values do not prove the practice of fertilization because most values come from modern specimens.

Terrestrial faunal remains (e.g., archaeological remains of wild species of birds, cervids, rodents and domesticated mammals such as *Lama* sp. and *Cavia porcellus*) show *δ*^13^C_coll_ and *δ*^15^N mean values ranging between -16.6 ± 3.2‰ and +7.3 ± 2.2‰, accordingly. The *δ*^13^C_coll_ values of these herbivorous species fall between the range of C_3_ and C_4_ plants and show a large variability relative to region and period (Figure SI 1). Their *δ*^15^N values are not fully consistent with water stress (*89-90*). The *δ*^13^C and *δ*^15^N mean values for marine fauna (including fishes, little fishes such as anchovy and sardines, mollusks, and marine birds and mammals from modern and archaeological origin) are -11.9 ± 1.9 ‰ and +13.8 ± 3.0 ‰, respectively, and were significantly different from terrestrial fauna (p = 0.000 for *δ*^13^C and *δ*^15^N). Freshwater fauna (mainly from Titicaca basin) has *δ*^13^C and *δ*^15^N mean values of -19.6 ± 2.5 ‰ and +7.7 ± 2.0 ‰.

**
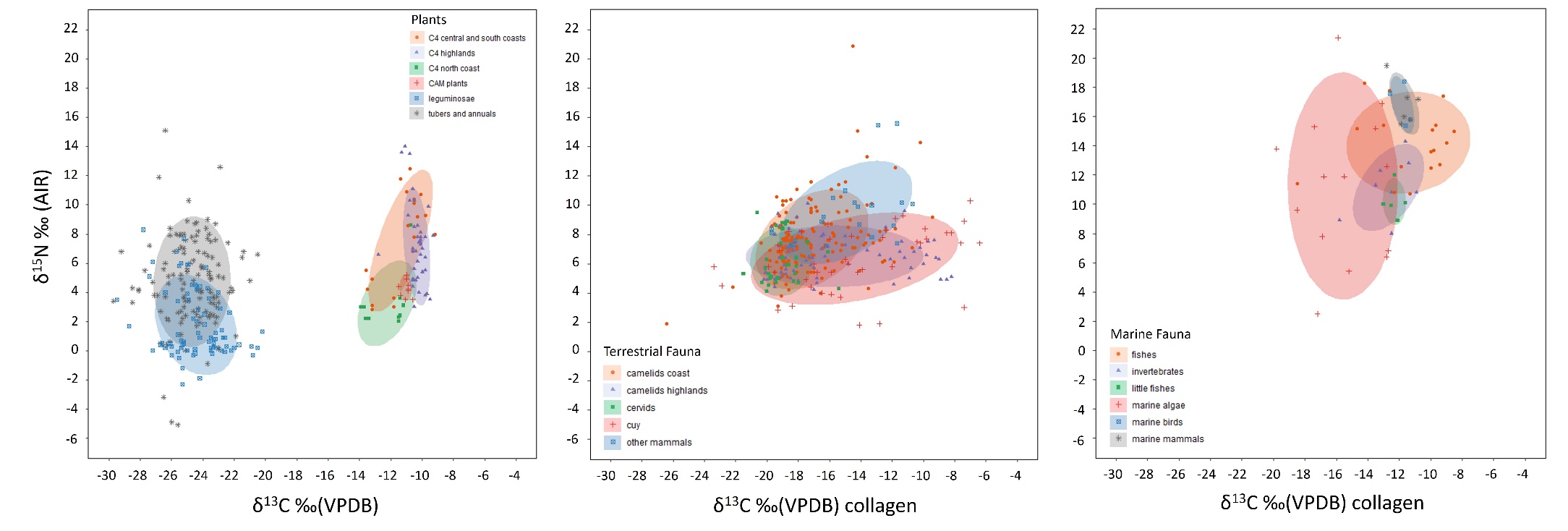
**

Figure SI3 1: Scatterplots of *δ*^13^C_coll_ and *δ*^15^N values and Standard Ellipse Areas for edible plants and fauna of the Central Andes discriminated by categories. Each Standard Ellipse Area of isotopic niches represent an estimated 68% of the population. The C_4_ plants show latitudinal differences (drived mainly by maize), whereas camelids’ values also show some regional variation.

**FRUITS** **models implementation (modified from Pezo-Lanfranco et al., 2018, 2022: SI 2; Colonese et al., 2020; Toso et al., 2021).**

The proportional contribution of different food sources to the human diet was estimated using a mixed Bayesian mixing model in FRUITS v. 2.1.1 program (Food Reconstruction Using Isotopic Transferred Signals: Fernandes, 2014; Fernandes et al., 2015), which uses dietary proxies (stable isotope values) of humans and food items and Bayesian calculation, to estimate the most likely values of the contribution of each type of food to the diet of individuals and populations.

FRUITS models were implemented according to the following Steps:

1. We choose a concentration-dependent and routing model using 10000 iterations (Fernandes, 2016). Then, the model was “loaded” with *dietary proxies:* δ^13^C_col_, δ^15^N_col_, and δ^13^C_ap_ (models of three proxies) or δ^13^C_col_, and δ^15^N_col_ (models of two proxies) **[see Figure SI3 2.1]**.
2. We add *consumer data:* for groups of individuals, we use the average isotope values of each group/phase and standard error of the mean; for individuals, we considered the isotope values with an uncertainty of ±0.5 (Fernandes et al., 2015) **[see Figure SI3 2.2]**.
3. We add four potential food sources presumably consumed (*Food groups*): terrestrial animals (providing proteins and lipids), called in the model “TF”; fish and mollusks (providing proteins and lipids), called “MF”; and C_3_ and C_4_ plants (providing carbohydrates and proteins), called C_3_ and C_4_, accordingly [see Figure SI3 2.3], and their respective composition of macronutrients (*Food fractions: bulk, protein, and energy*) [see Figure SI3 2.4].
4. Isotope fractionation factors (*offsets*), sourced from Fernandes et al. (2015), were established from the consensual values derived from experimental studies [see Figure SI3 2.5]. For *δ*^13^C was set at +4.1 ± 0.5‰ between diet and collagen, and +10.1 ± 0.5 ‰ between diet and apatite; and the *δ*^15^N diet-collagen fractionation factor was set at +5.5 ± 0.5 ‰. The *weighted values* of each *food fraction* were set based on the parameters published by Fernandes et al. (2015). The values of *δ*^13^C_ap_ represent the total carbon (100%) in the diet (Ambrose and Norr, 1993). For collagen, we assumed that nitrogen was derived exclusively from proteins (100%), whereas carbon can be routed from carbohydrates and lipids recycled during the synthesis of non-essential amino acids (Ambrose and Norr, 1993). Therefore, the carbon from the protein and energy routed to the total collagen was established at 74 ± 4% and 26%, respectively (Fernandes et al., 2012). Lipids and carbohydrates were added to the model as “energy.”
5. For each *food group*, the mean *δ*^13^C_col_*,* and *δ*^15^N values of each *food fraction* (protein, carbohydrates, and lipids) were estimated using the fractionation values suggested by previous reconstructions using FRUITS (Fernandes et al., 2015; Fernandes, 2016): −2‰ (*∆*^13^C_protein-collagen_), −8‰ (*∆*^13^C_lípids-collagen_), and +2‰ (∆^15^N_protein-collagen_) for terrestrial mammals and −1‰ (*∆*^13^C_protein-collagen_), −7‰ (∆^13^C_lípids-collagen_), and +2‰ (∆^15^N_protein-collagen_) for marine animals. For plants, the offsets were −2‰ (∆^13^C_bulk-protein_) and +0.5‰ (∆^13^C_bulk-lipids_), whereas that for the *δ*^15^N value of plant protein, the value of δ^15^N recorded for the plant was assumed. The standard deviation of *δ*^13^C_col_*,* and *δ*^15^N values was used as uncertainty factor **[see Figure SI3 2.6]**.
6. The carbon weight (*concentrations*) of each food fraction (protein and energy) from each food group was calculated according to its nutrient composition as per the methods described by Fernandes et al. (2015: 329, SI Table 1; see also Fernandes et al. 2014: 4-6 for calculation methods). The nutrient content (protein, carbohydrates/lipids) of the food groups is relatively constant and expressed as dry weight carbon content (wtC%). The macronutrient content for each food group was set as follows: C_4_ plants (protein: 7% ± 2.5% wtC%, carbohydrates/lipids: 93% ± 2.5% wtC%), C_3_ plants (protein: 5% ± 2.5% wtC%, carbohydrates/lipids: 95% ± 2.5% wtC%), meat of terrestrial animals (30% ± 2.5% wtC%, carbohydrates/lipids: 70% ± 2.5% wtC%), and meat of marine animals (65% ± 5% wtC %, carbohydrates/lipids: 35% ± 5% wtC%) **[see Figure SI3 2.7]**.
7. A conservative, and physiologically acceptable, range of protein consumption was stipulated between 5% and 45% of total calories (Fernandes et al., 2014). This input was charged as “prior” **[see Figure SI3 2.8].**
8. Based on these considerations and guided by our observations of the bivariate models (δ^15^N vs. δ^13^Ccol; δ^13^Ccol vs. δ^13^Cap), several alternative models with different priors corresponding to different dietary scenarios were tested:
   - 1. Neutral model, no priors.
     2. Assuming predominance of marine fauna in the diet.
     3. Assuming more maize than marine/terrestrial fauna in the diet.
     4. Assuming more terrestrial fauna than other resources in the diet.
9. Then, we select the model that better fits with the expected context inferred from the archaeological record.

**Parameters for FRUITS models (inputs)**

Figure SI3 2.1
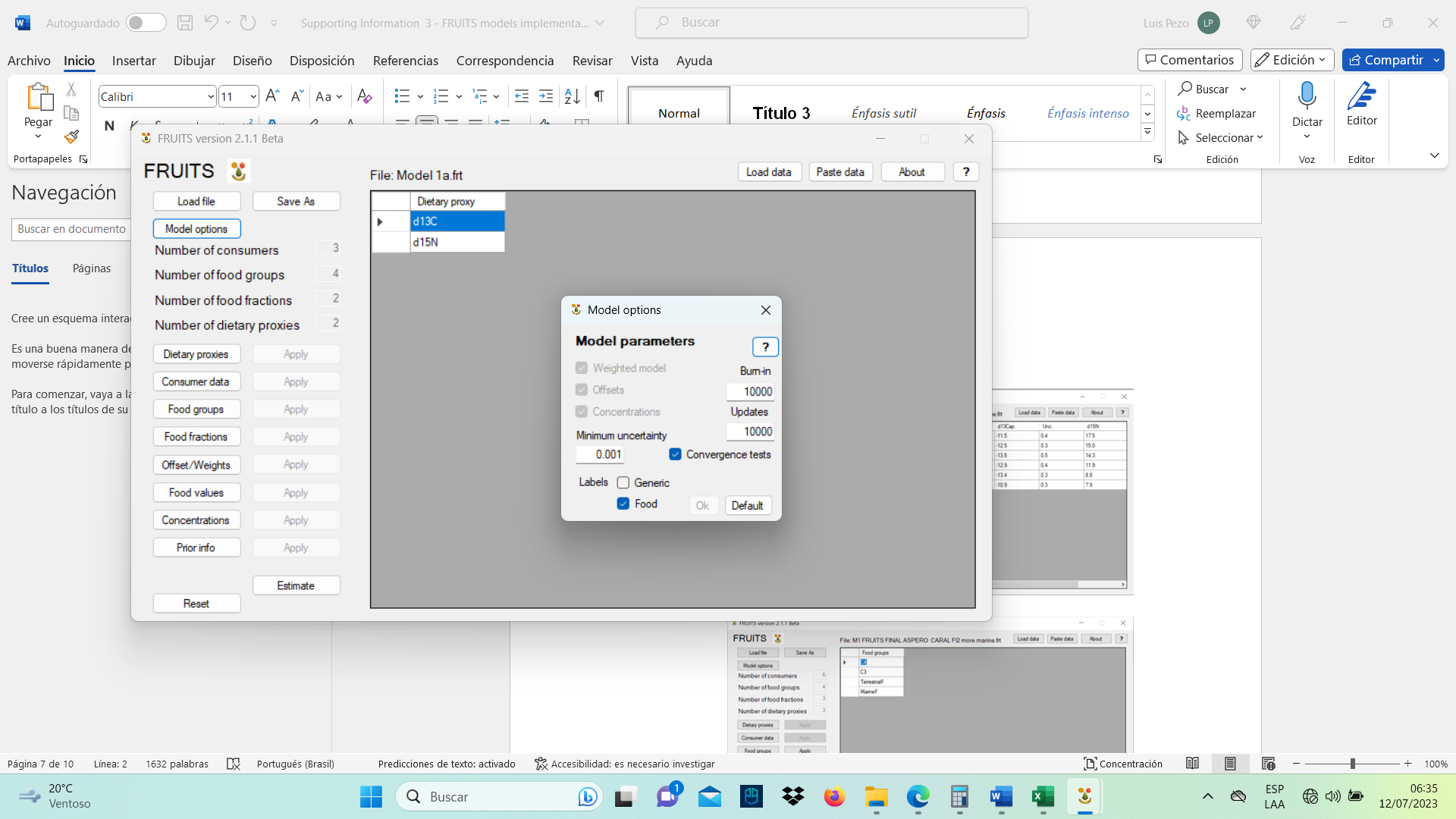


Figure SI3 2.2
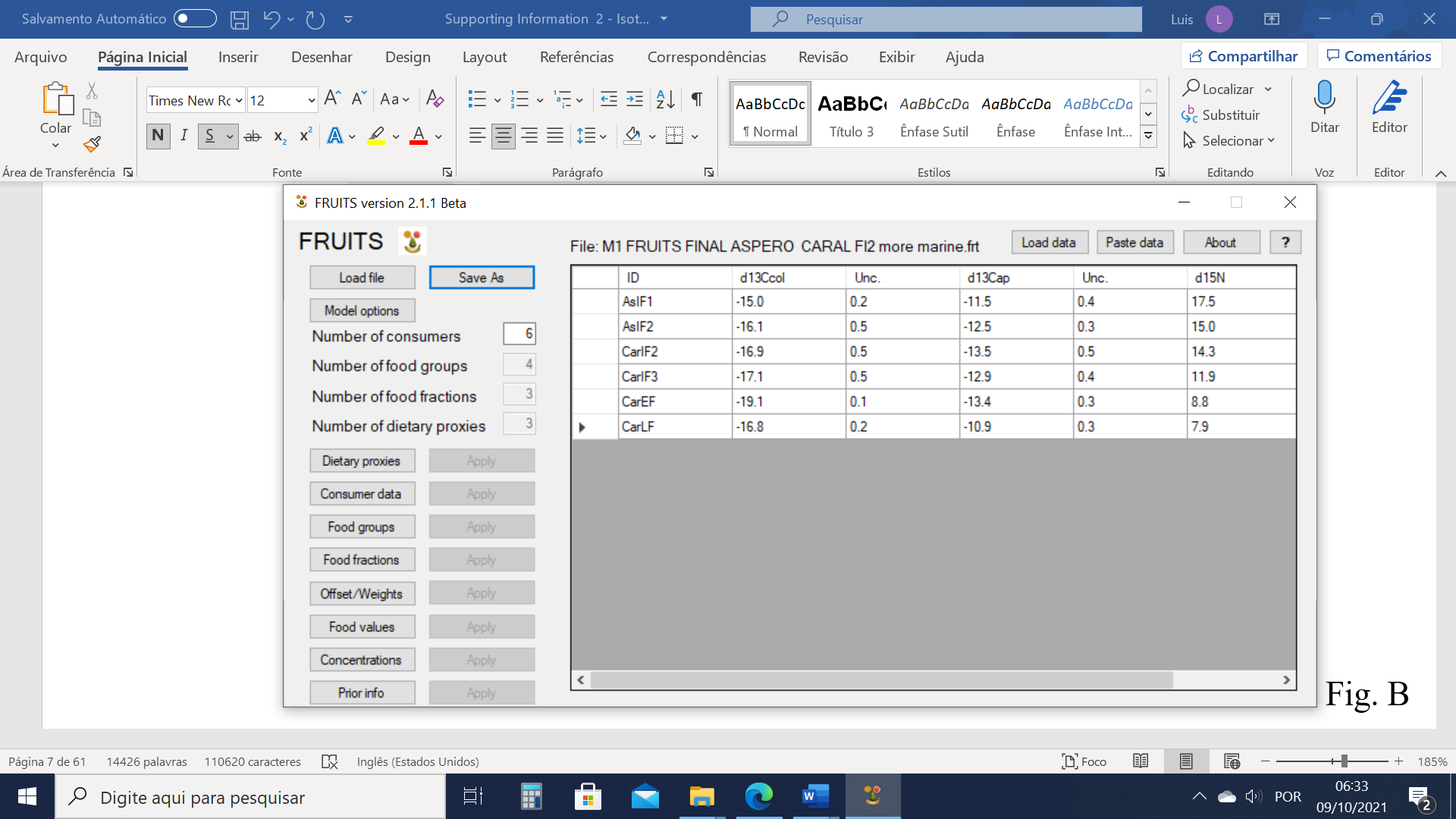


Figure SI3 2.3
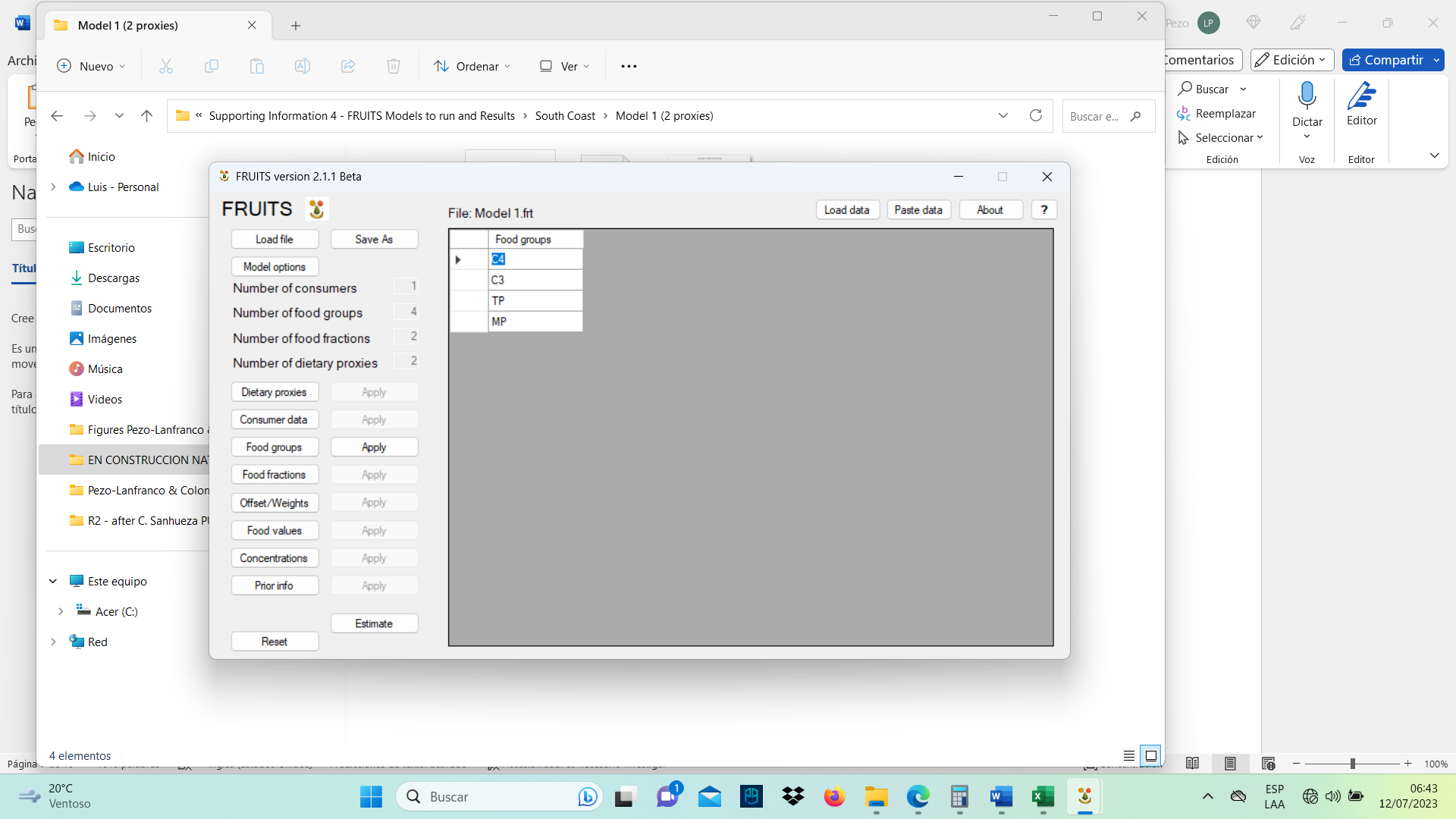


Figure SI3 2.4
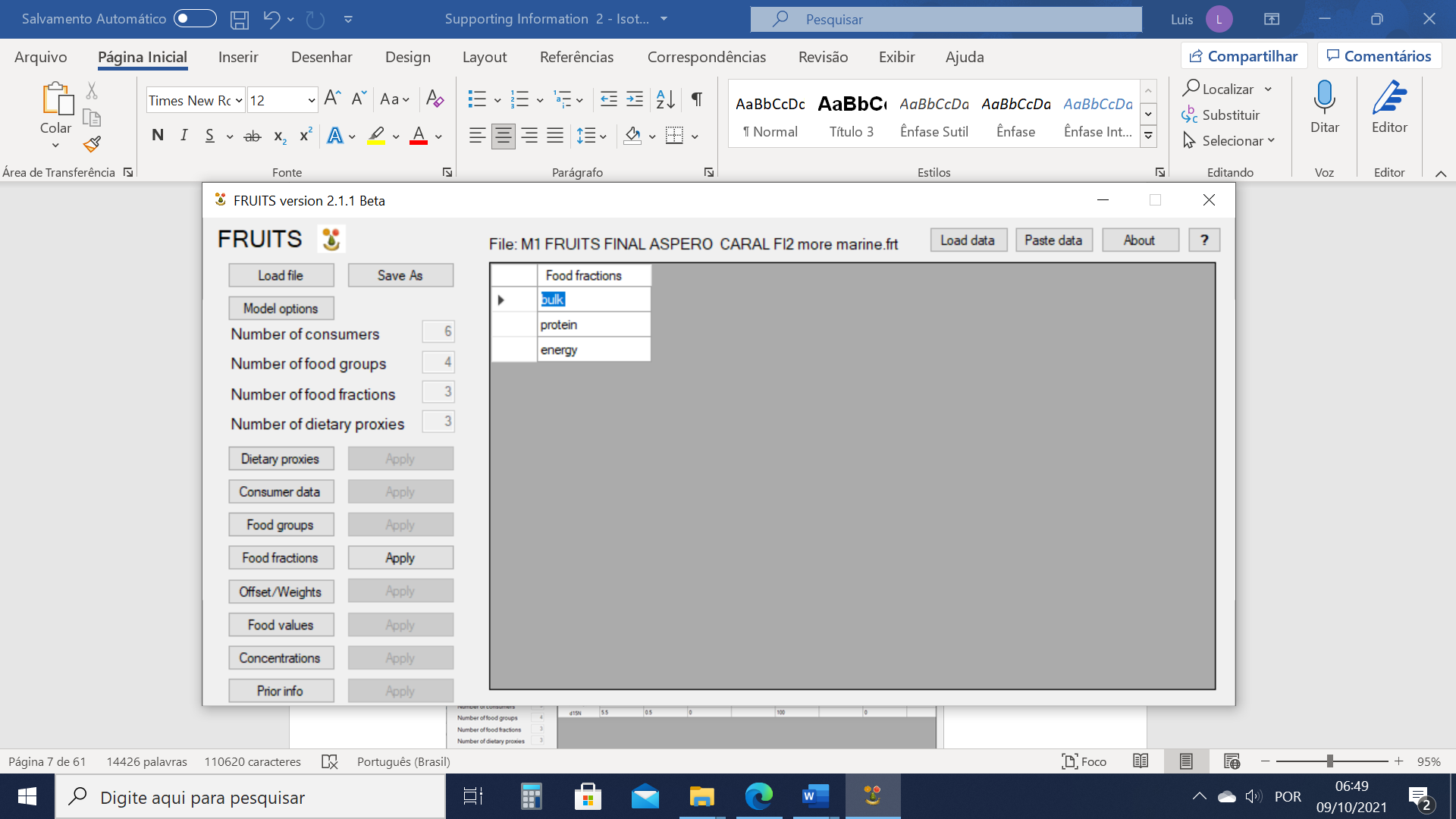


Figure SI3 2.5
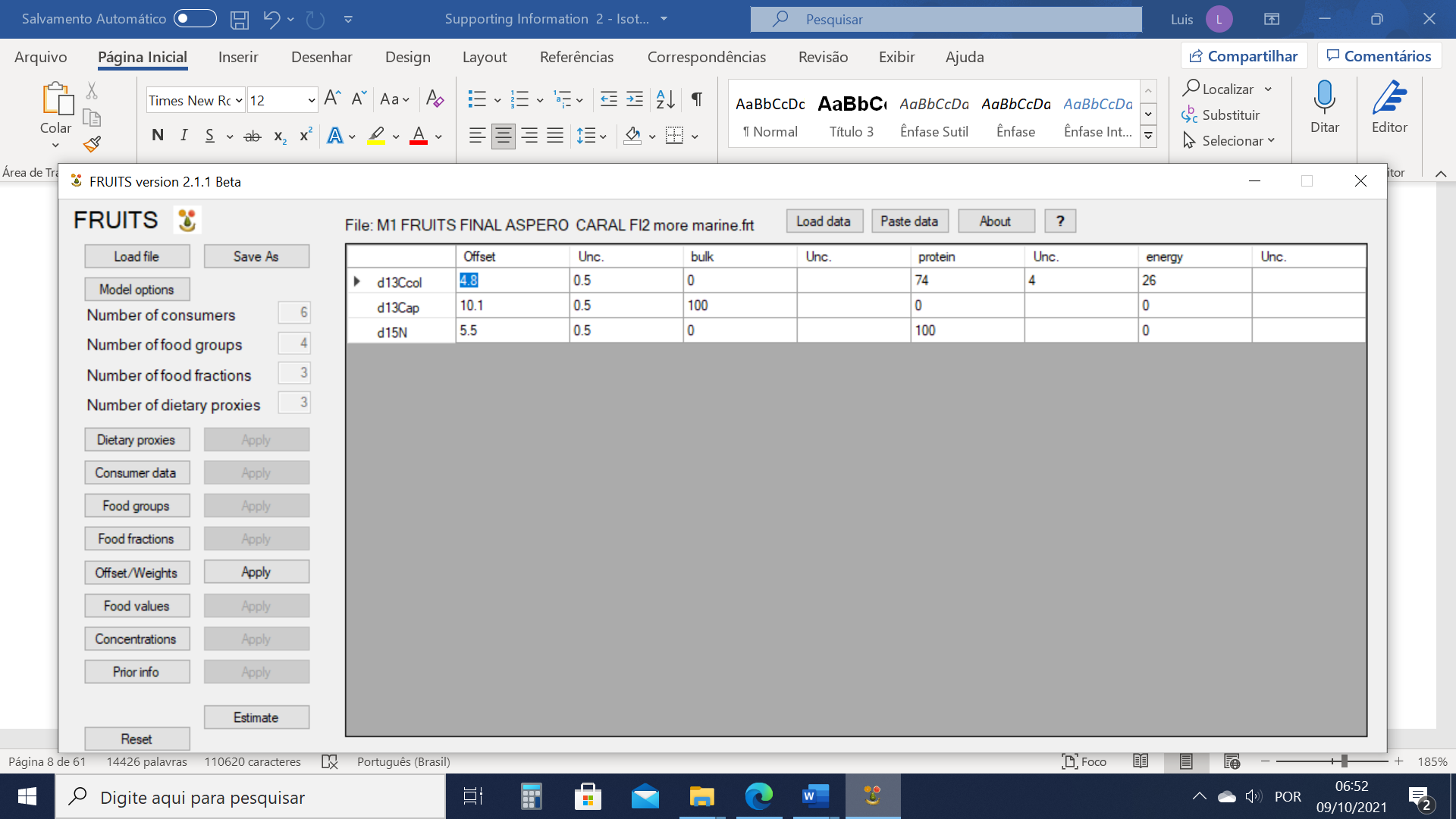


Figure SI3 2.6
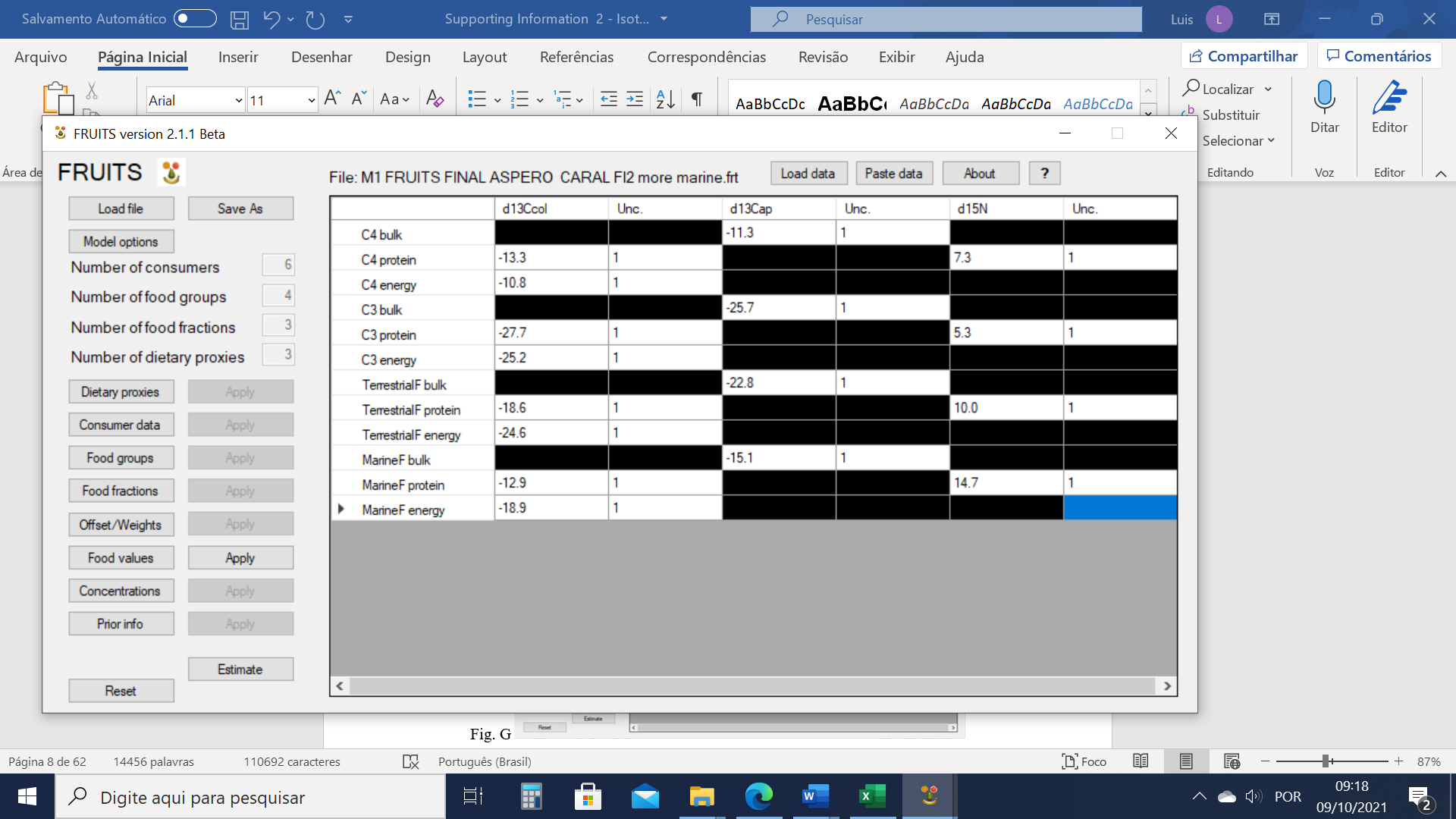


Figure SI3 2.7
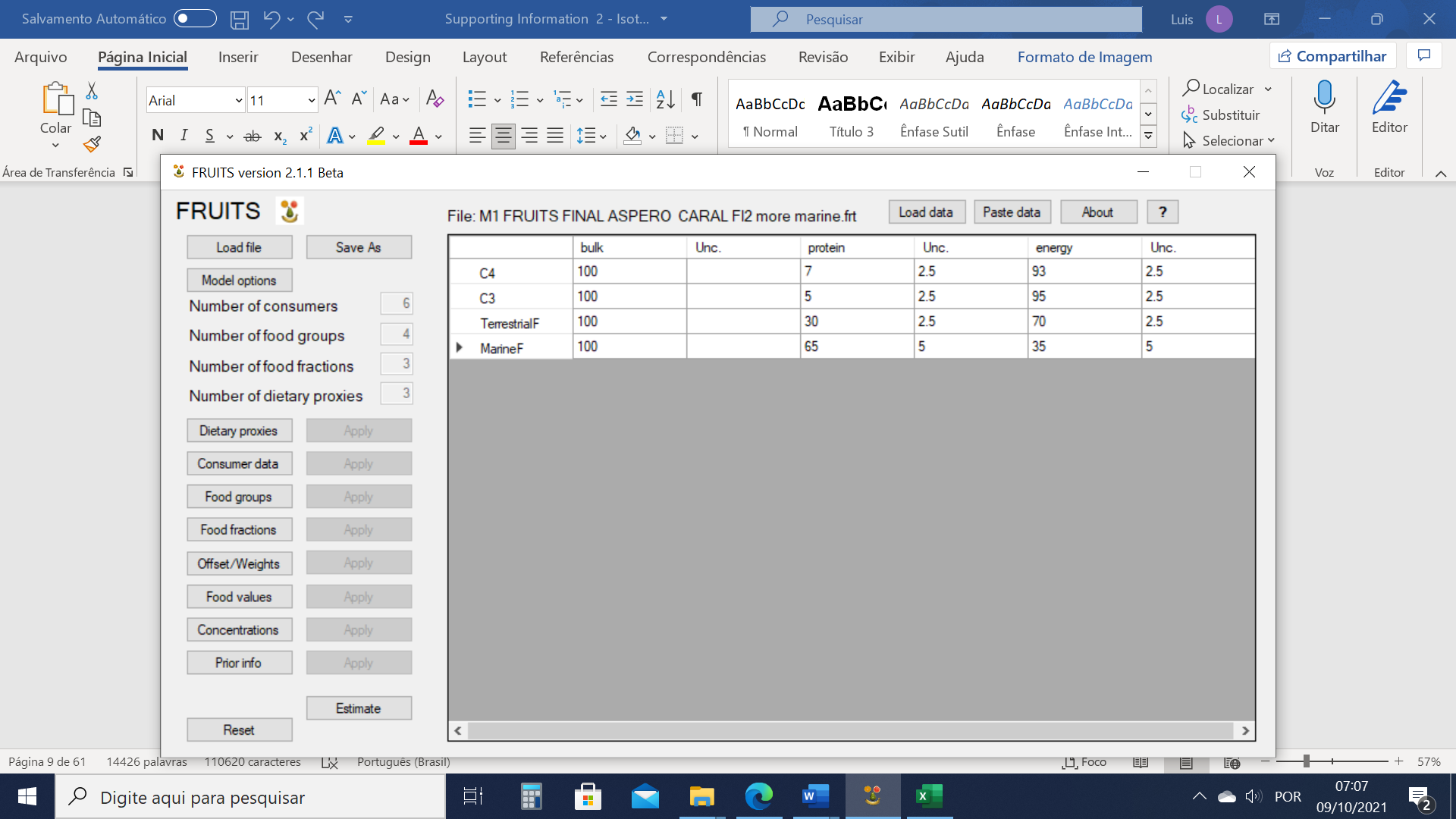


Figure SI3 2.8: Examples of FRUITS models whit different priors

| 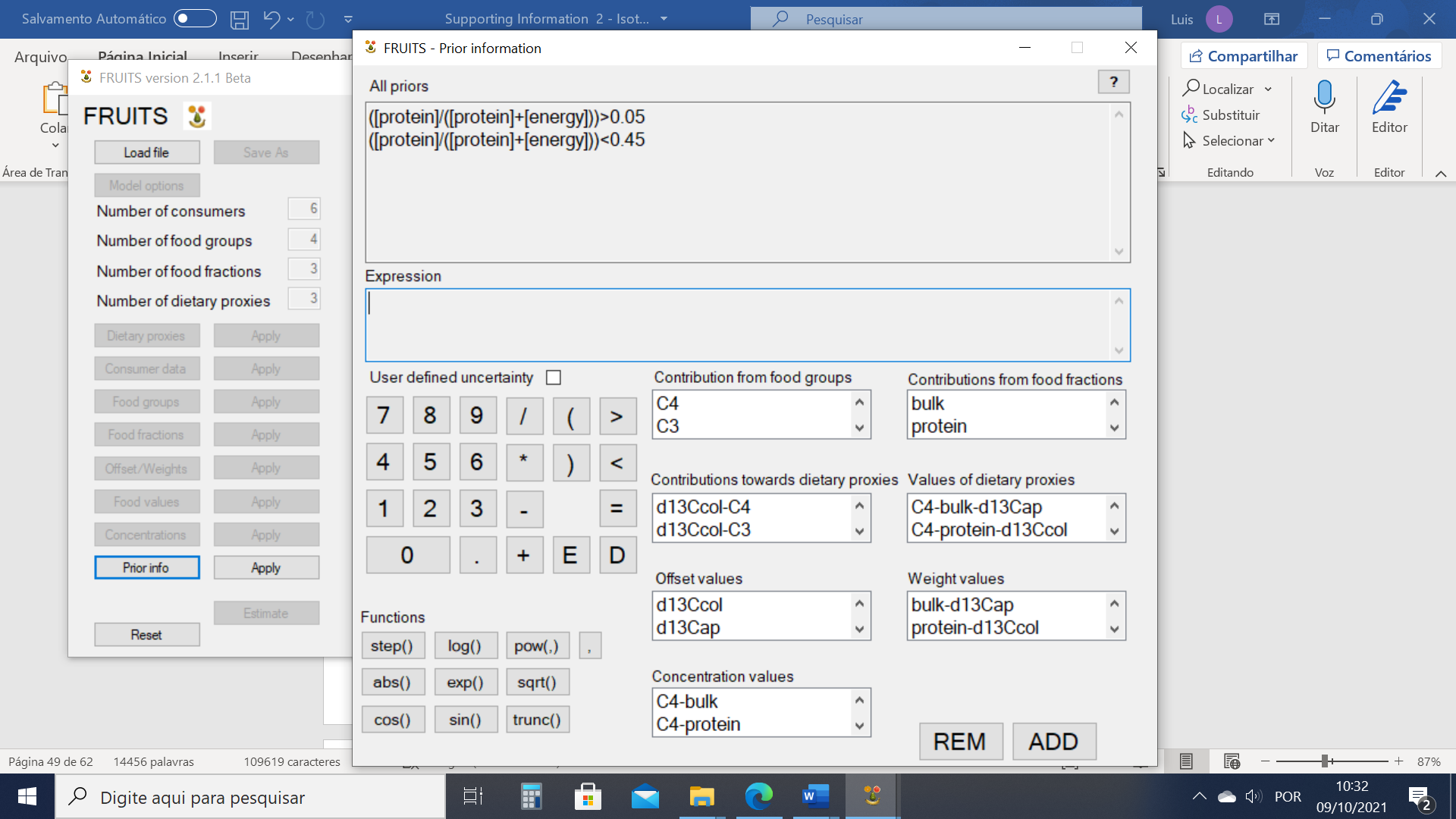 | 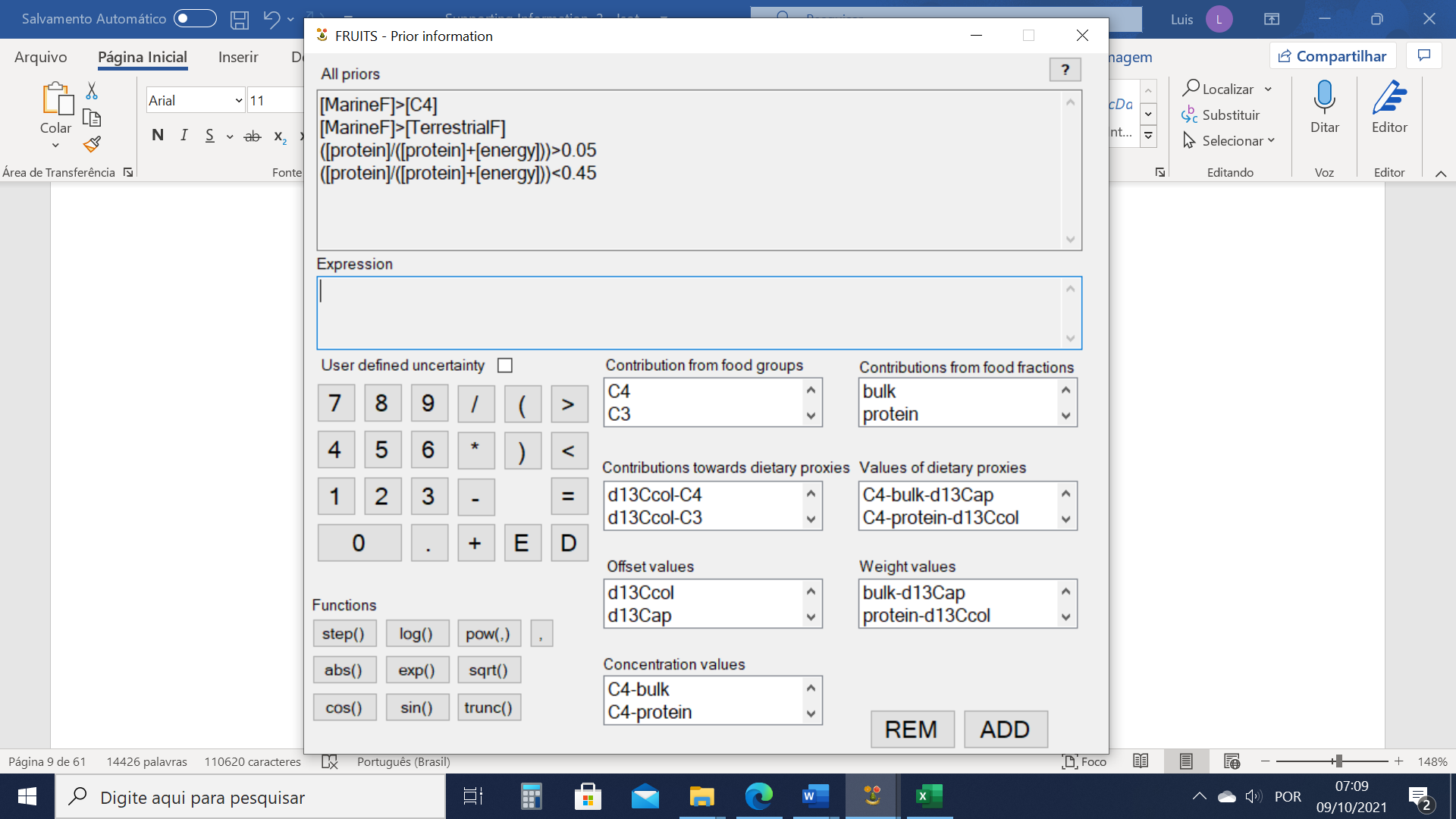 |
| --- | --- |
| Neutral model, no priors other than physiologic limits | Assuming predominance of marine fauna in the diet |

| 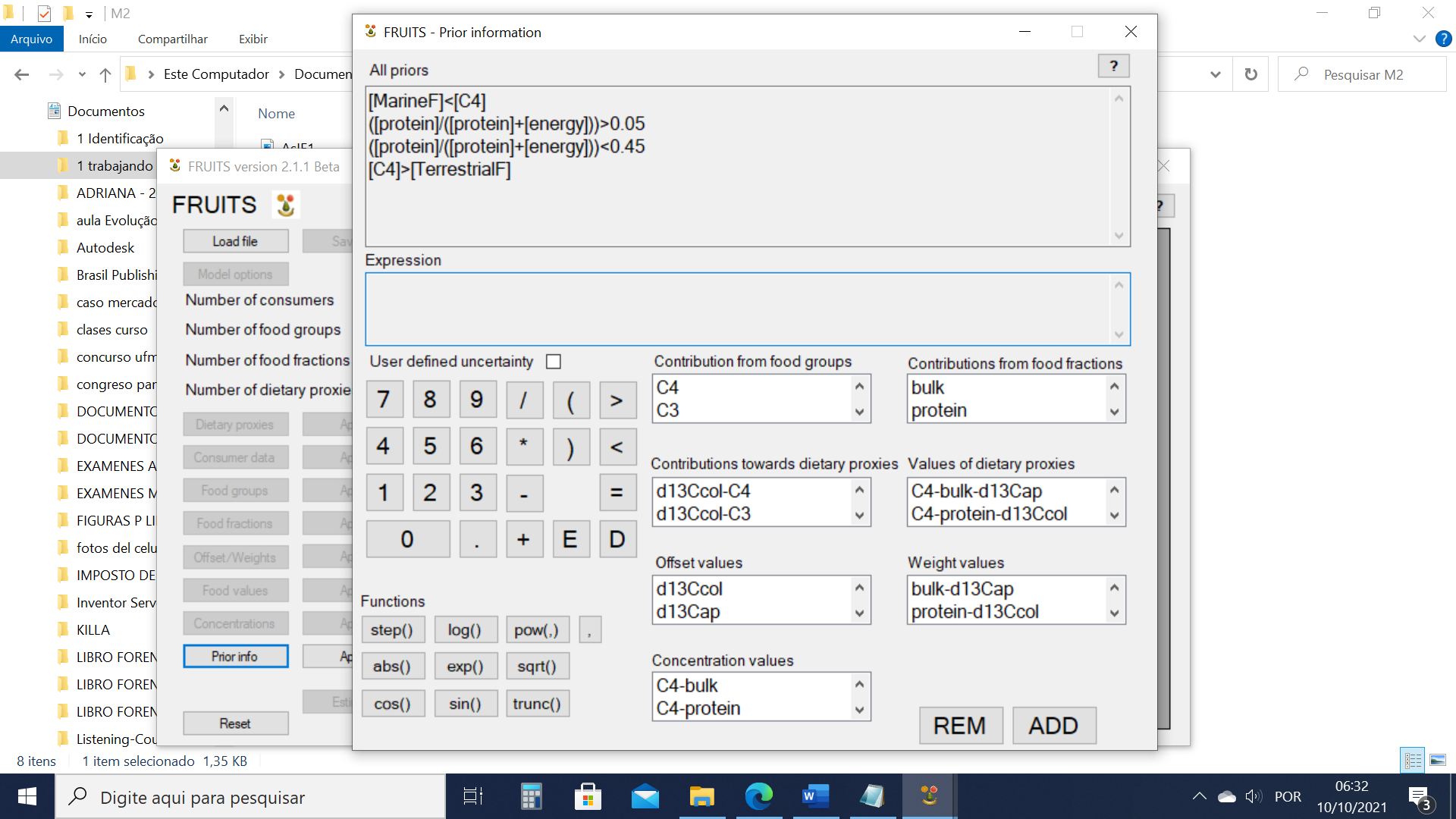 | 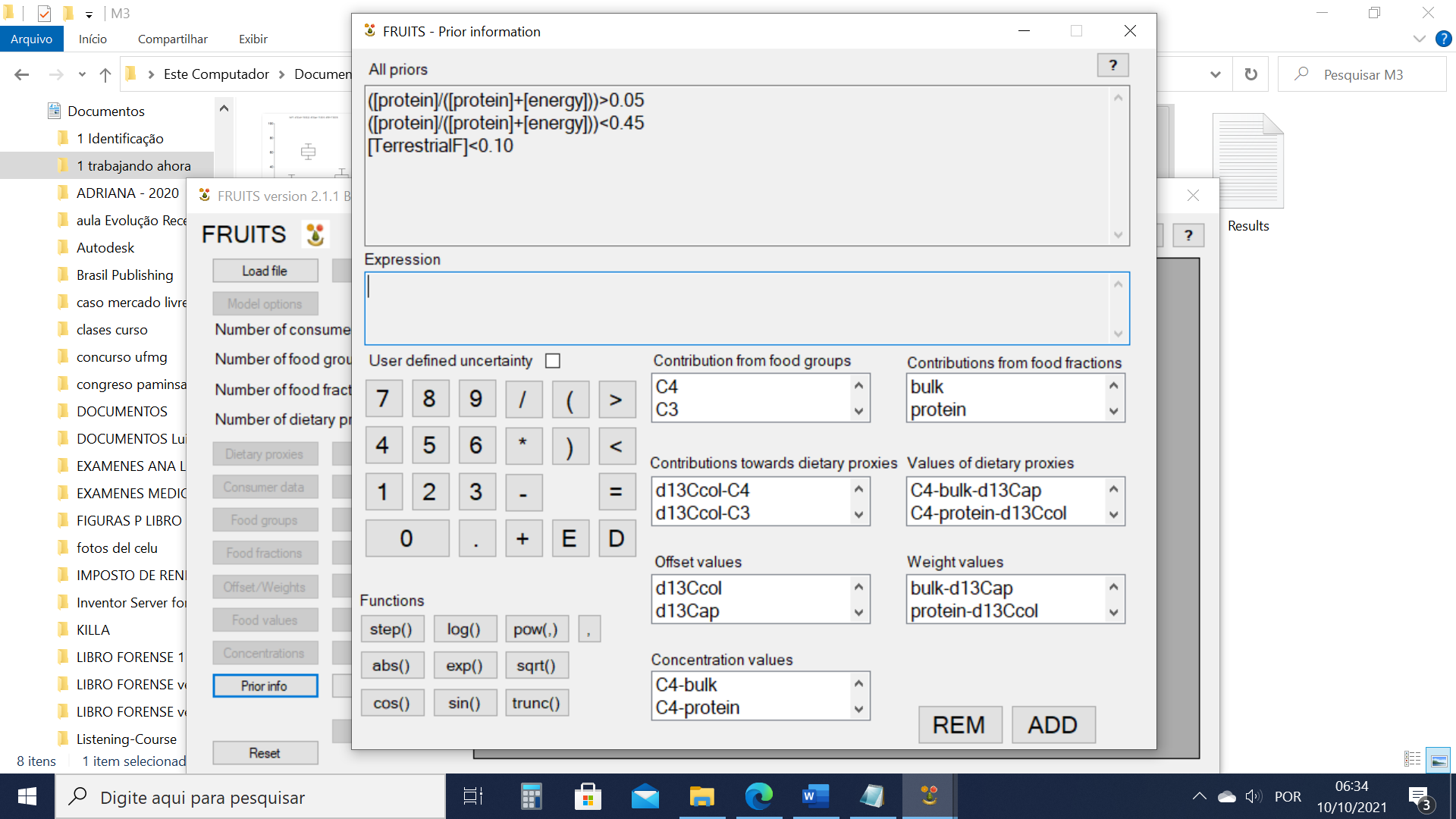 |
| --- | --- |
| Assuming more maize than marine /terrestrial fauna in the diet | Assuming terrestrial fauna consumption < 10% in the diet |

**Summary of general input data**

**INPUT DATA** (Weighted model: Yes; Offset dependent: Yes; Concentration-dependent model: Yes; Minimum uncertainty: 0.001)

**Offsets**

[d13Ccol]=4.8(0.5) [d13Cap]=10.1(0.5) [d15N]=5.5(0.5)

**Weights**

[d13Ccol][bulk]=0() [d13Ccol][protein]=74(4) [d13Ccol][energy]=26()

[d13Cap][bulk]=100() [d13Cap][protein]=0() [d13Cap][energy]=0()

[d15N][bulk]=0() [d15N][protein]=100() [d15N][energy]=0()

**Concentrations**

[C4][bulk]=100() [C4][protein]=7(2.5) [C4][energy]=93(2.5)

[C3][bulk]=100() [C3][protein]=5(2.5) [C3][energy]=95(2.5)

[TerrestrialF][bulk]=100() [TerrestrialF][protein]=30(2.5) [TerrestrialF][energy]=70(2.5)

[MarineF][bulk]=100() [MarineF][protein]=65(5) [MarineF][energy]=35(5)

**Common Prior info**

([protein]/([protein]+[energy]))>0.05

([protein]/([protein]+[energy]))<0.45

**Source/Food values:** See Supplemental Information 1 for raw and summary data.

**Group/Individual values:** See Supplemental Information 2 for raw and summary data.

**References**

1. Heaton, T. et al., Marine20 - the marine radiocarbon age calibration curve (0–55,000 cal BP). *Radiocarbon* **62**, 779–820 (2020).
2. Hogg, A. et al., SHCal20 southern hemisphere calibration, 0–55,000 years cal BP. *Radiocarbon* **62**, 759–778 (2020).
3. Bronk Ramsey, C. Bayesian analysis of radiocarbon dates. *Radiocarbon* **51**, 337–360 (2009).
4. P. J. Reimer, R. W. Reimer, A marine reservoir correction database and on-line interface. *Radiocarbon* **43**, 461–463 (2001).
5. Marino, B. D. & McElroy, M. B. Isotopic Composition of Atmospheric CO2 Inferred from Carbon in C4 Plant Cellulose. *Nature* **349**, 127–131 (1991).
6. Albeke, S. E. rKIN:(kernel) isotope niche estimation. R package version 0.1 (2017).
7. Robinson, J.R. Investigating Isotopic Niche Space: Using rKIN for Stable Isotope Studies in Archaeology. *J. Archaeol. Method Theory* 29, 831–861 (2022).
8. Pezo-Lanfranco, L. et al. Middle Holocene plant cultivation on the Atlantic Forest coast of Brazil? *R. Soc. Open Sci.* **5**, 180432 (2018).
9. Pezo-Lanfranco, L., Machacuay, M., Novoa, P., Peralta, R., Mayer, E., Eggers, S., & Shady, R. The diet at the onset of the Andean Civilization: New stable isotope data from Caral and Aspero, North-Central Coast of Peru. *Am. J. Biol. Anthropol.* **177**, 402–424 (2022).
10. Colonese, A. C., Winter, R., Brandi, R., Fossile, T., Fernandes, R., Soncin, S., McGrath, K., Von Tersch, M. & Bandeira, A. M. Stable isotope evidence for dietary diversification in the pre-Columbian Amazon. *Sci. Rep.* **10**, 16560 (2020).
11. Toso, A. et al., Fishing intensification as response to Late Holocene socio-ecological instability in southeastern South America. *Sci. Rep.* 11, 23506 (2021).
12. Fernandes, R., Millard, A. R., Brabec, M., Nadeau, M. J., & Grootes, P. Food reconstruction using isotopic transferred signals (FRUITS): A Bayesian model for diet reconstruction. *PLoS One* **9(2)**, e87436. (2014).
13. Fernandes, R., Grootes, P. M., Nadeau, M. J., & Nehlich, O. Quantitative diet reconstruction of a neolithic population using a Bayesian mixing model (FRUITS): The case study of Ostorf (Germany). *Am. J. Phys. Anthropol.* **158**, 325–340 (2015).
14. Fernandes, R. *FRUITS Manual*. 2nd edn. https://sourceforge. net/projects/fruits/ (2016)
15. Ambrose, S. H., & Norr, L. Experimental evidence for the relationship of carbon isotope ratios of whole diet and dietary protein to those of bone collagen and carbonate in *Prehistoric Human Bone: Archaeology at the Molecular Level* (eds. Lambert, J. & Grupe, G.) 1–37 (Springer, Berlin, 1993).
16. Fernandes, R., Nadeau, M. J., & Grootes, P. M. Macronutrient-based model for dietary carbon routing in bone collagen and bio-apatite. *Arch. Anthropol. Sci.* **4**, 291–301 (2012).
